# Supplementary material for: Nanodiamond Effects on Cancer Cell Radiosensitivity: The Interplay between Their Chemical/Physical Characteristics and the Irradiation Energy
Source: Int J Mol Sci. 2023 Nov 22;24(23):16622. doi: 10.3390/ijms242316622 (PMC10706717; doi:10.3390/ijms242316622)
Supplement: Supplementary file 1 [file ijms-24-16622-s001.zip › ijms-2665125-supplementary.pdf]

SUPPLEMENTARY MATERIALS

Nanodiamond effects on cancer cell radiosensitivity: the interplay between their chemical/physical characteristics and the irradiation energy

Veronica Varzi [1,2,†], Emiliano Fratini [2,†], Mauro Falconieri [3], Daniela Giovannini [2], Alessia Cemmi [4], Jessica Scifo [4], Ilaria Di Sarcina [4], Pietro Aprà [1], Sofia Sturari [1], Lorenzo Mino [5], Giulia Tomagra [6], Erminia Infusino [7], Valeria Landoni [7], Carmela Marino [2], Mariateresa Mancuso [2], Federico Picollo [1,*], Simonetta Pazzaglia [2,*]

[1] Physics Department, NIS Inter-departmental Centre, University of Turin and National Institute of Nuclear Physics, Section of Turin, Via Giuria 1, 10125 Turin, Italy.

[2] Laboratory of Biomedical Technologies, Italian National Agency for New Technologies, Energy and Sustainable Economic Development (ENEA), Casaccia Research Centre, Via Anguillarese 301, 00123 Rome, Italy.

[3] Fusion and Technology for Nuclear Safety and Security Department, ENEA, Casaccia Research Centre, Via Anguillarese 301, 00123 Rome, Italy.

[4] Innovative Nuclear Systems Laboratory, ENEA, Casaccia Research Center, Via Anguillarese 301, 00123 Rome, Italy.

[5] Department of Chemistry and NIS Inter-departmental Centre, University of Turin, Via Giuria 7, 10125 Turin, Italy.

[6] Drug Science and Technology Department, University of Turin, Corso Raffaello 30, 10125 Turin, Italy.

[7] Medical Physics Laboratory, IRCCS Istituto Nazionale Tumori Regina Elena, Via Elio Chianesi 53, 00144 Rome, Italy.

* Correspondence: federico.picollo@unito.it (F.P.); simonetta.pazzaglia@enea.it (S.P.); Tel.: +39-011-670-7879 (F.P.); +39-06-3048-6535 (S.P.); Fax: +39-011-670-7020 (F.P.); +39-06-3048-3644 (S.P.)

† These authors contributed equally to this work.

Supplementary materials

Supplementary figures. S1-S6

Supplementary Figure S1

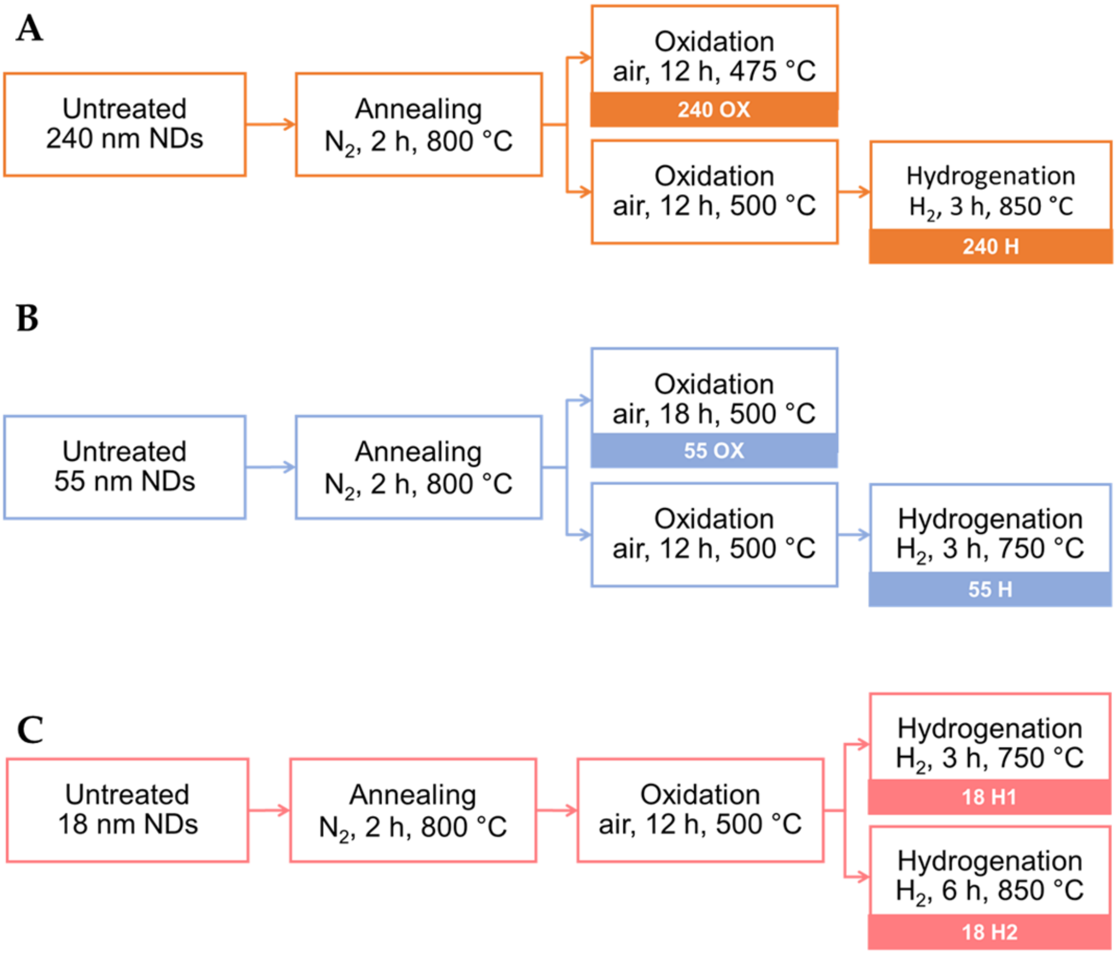

S1. Summary schemes for the thermal treatments performed on NDs.
All the nanodiamonds (NDs) under study were subjected to thermal treatments to modify their surface. The various samples have been subjected to different processing steps, which are summarized in the schemes of the figure, which also report the labels chosen to indicate each batch of NDs. **(A)** Thermal treatments carried out on 240 nm NDs. **(B)** Thermal treatments conducted on 55 nm NDs. **(C)** Thermal treatments performed on 18 nm NDs. The names of the processes were reports inside the white rectangles, whereas their sequence was indicated by the arrows. The names given to the samples were reported inside the colored rectangles.

Supplementary Figure S2

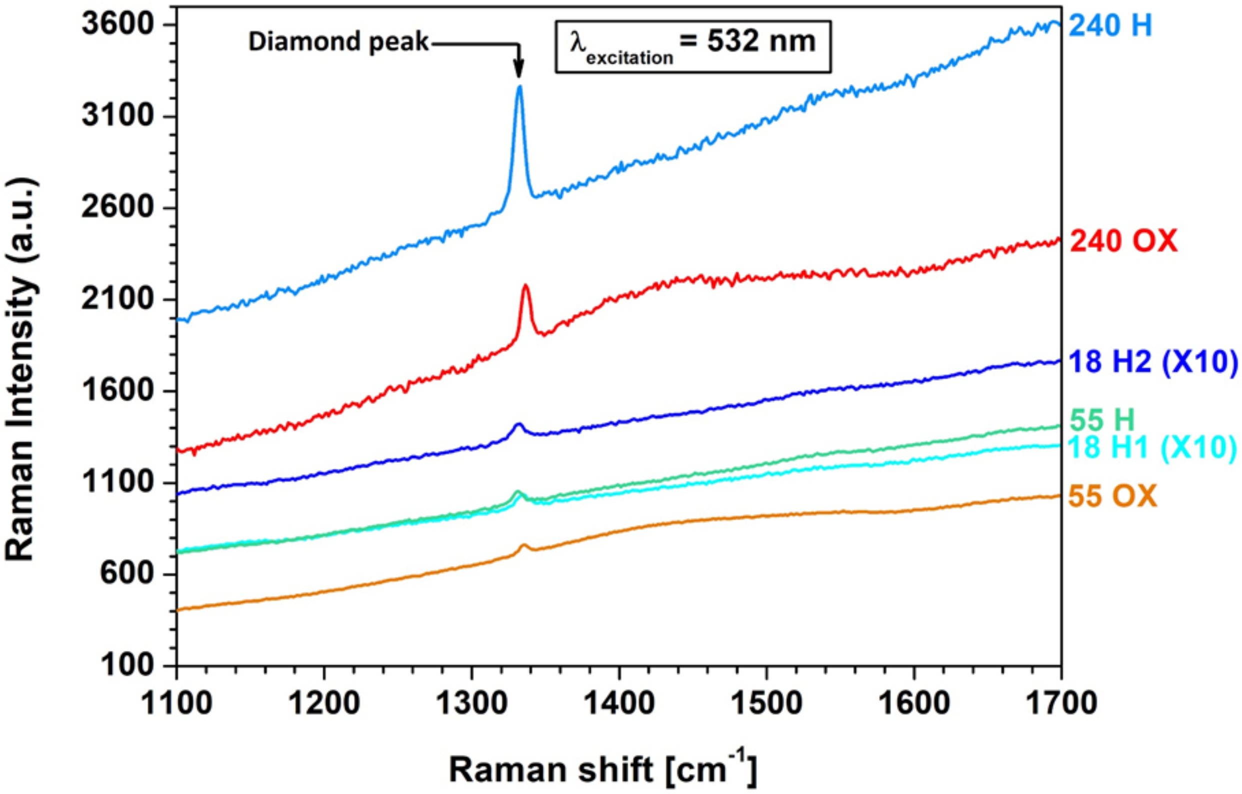

S2. Raman spectra of the processed NDs.

Raman spectroscopy was performed to investigate the structure of the NDs subjected to oxidation and hydrogenation thermal treatments (OX- and H-NDs respectively). The figure reports Raman spectra of the samples, where the only evident feature is the first-order Raman peak of diamond at 1332 cm-1 [24], which evidences that the processes in air and hydrogen atmosphere did not undermine ND diamond core. On the other hand, Raman spectra do not present G-band at 1580 cm-1 [24] for any of the samples. This is probably related to the dominant photoluminescence (PL) signal due to NV centers, preventing the identification of a distinct peak associated with graphite. Nevertheless, it is possible to draw some conclusion related to sp2 phases, thanks to the information coming from PL spectra, displayed in Figure 1B in the main text. As examined in the comment of PL spectra, the collected PL data revealed a significant difference between the NDs of varying sizes in terms of luminescence intensity. This turned out to be two-orders-of-magnitude lower in 18 nm NDs with respect to 240 nm NDs. If, on one hand, such observation can be elucidated by considering the distinct dimensions of the diamond core of the NDs, on the other one, the observed phenomenon can also be associated with a more relevant presence of surface graphite on smaller NDs, in accordance with the findings by Smith et al. [59], who demonstrated that surface graphitic content has a quenching effect on the luminescence of NV centers. Thus, the reduced PL of smaller NDs may be attributed to the combination of factors, i.e., their smaller diamond core size and the potentially higher presence of graphitic phases on their surface. In conclusion, the insights gleaned from ND PL spectra support the hypothesis of a stronger presence of sp2 carbon in smaller-sized NDs compared to their larger counterparts. The spectra labelled as “18 H2” and “18 H1”, which are referred to the 18 nm NDs, were multiplied by 10 to render them visible on the same scale of the other ones.

Supplementary Figure S3

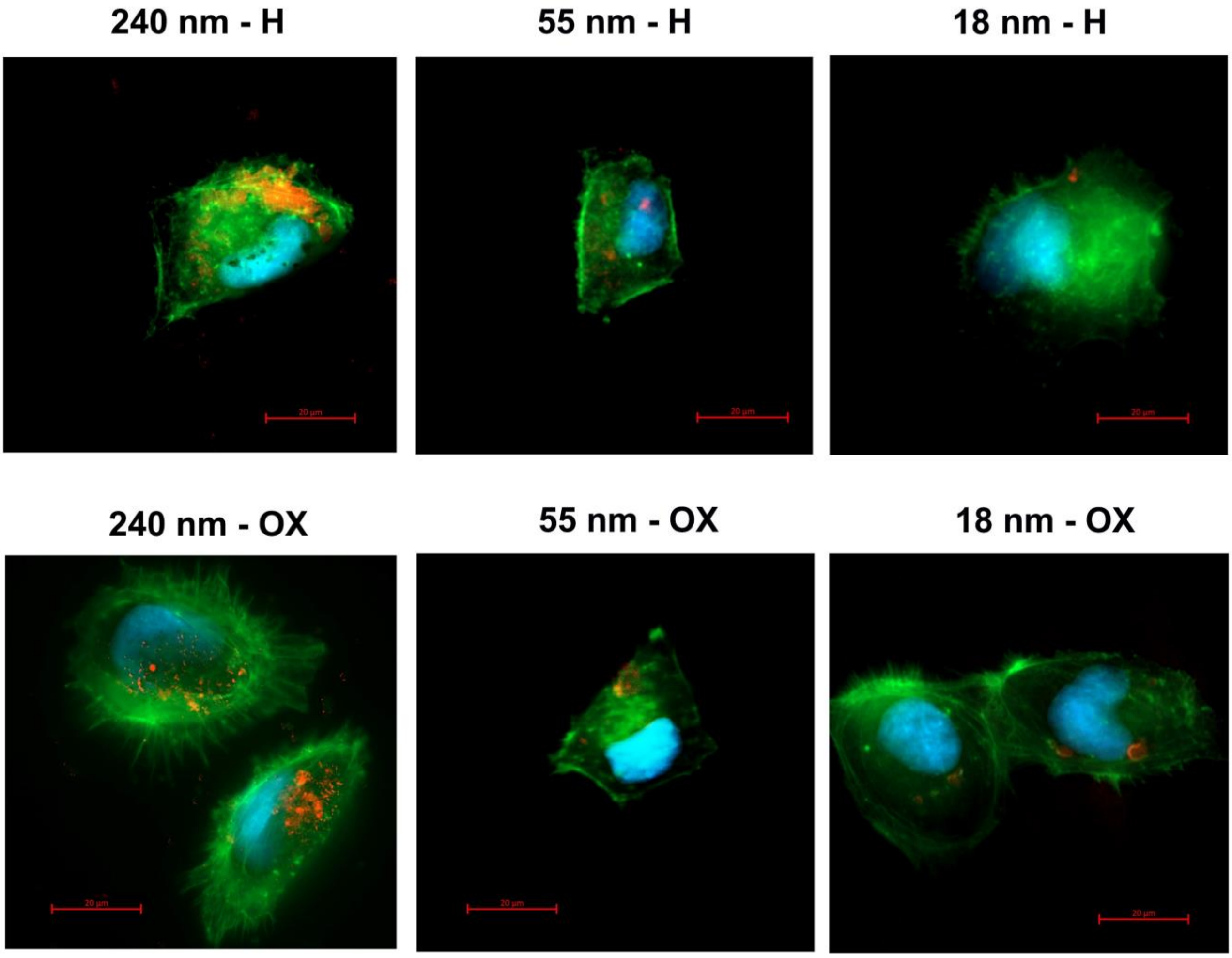

S3. Additional images of intracellular localizations of OX- and H-NDs.

Qualitative analysis of the cellular uptake of NDs by human DAOY cells through fluorescence microscopy: Comparison of DAOY cells after 13 h incubation with H- and OX- NDs of different sizes. Scale bar is 20 µm. The fluorescence imaging technique has been used to qualitatively analyze the internalization and localization of NDs in human MB DAOY cells by means of Red- Far red- ND emission (600-780 nm) upon 532 nm excitation. The cytoplasmic localization of NDs has been highlighted staining the cytoskeletal component F-actin (Phalloidin staining, green) and the cell nuclei (DAPI DNA staining, blu). As more appreciable in images of cells treated with 240 nm NDs, oxygenated NDs are more evenly dispersed throughout the cytoplasm, while hydrogenated NDs exhibit a notable tendency to cluster. The lower NDs signal for 55 nm and 18 nm NDs is due to the dependence of the fluorescence intensity on the ND size and not on difference in ND uptake or localization as pointed out in figures 1B, 2A, 2B, and S4.

Supplementary Figure S4

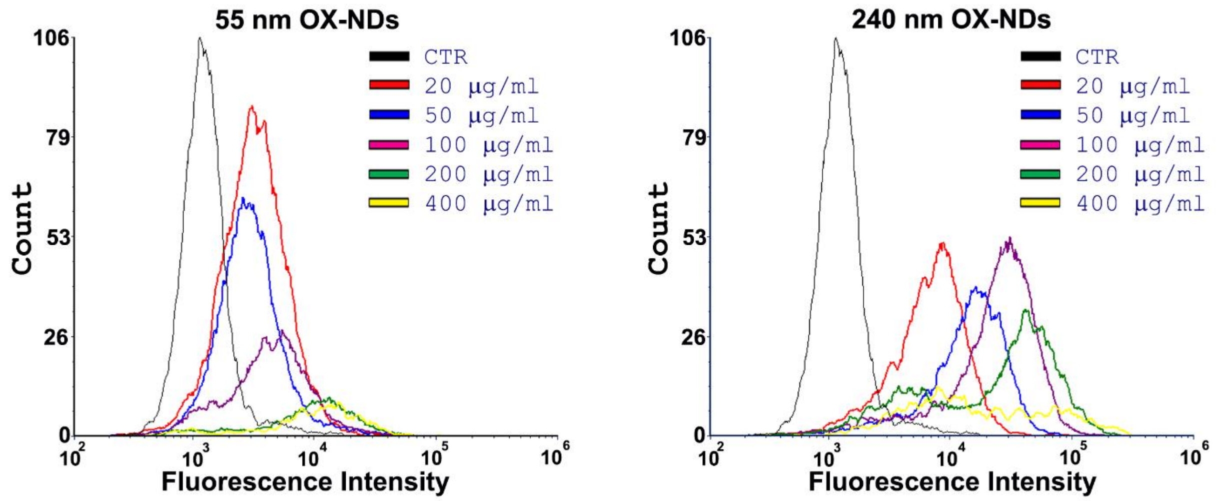

S4. Flow cytometry analysis for MB DAOY cells treated with different concentrations of 55 nm and 240 nm OX-NDs. We have analyzed the influence of ND concentration on their cell internalization. As shown in figure 2C, 55 nm H-NDs internalization is proportional to their concentration reaching a maximum at the highest concentration (400 μg/ml). The same analysis performed on 55 nm and 240 nm OX-NDs showed similar results with 240 OX-NDs that discerned variation even at low concentration (20 and 50 μg/ml) due to their higher intrinsic fluorescence. CTR, black; 20 μg/ml, red; 50 μg/ml, blue; 100 μg/ml, purple; 200 μg/ml, green; 400 μg/ml, yellow. Fluorescence intensity in the 712/20 channel is reported in the x-axis while the number of events in the y-axis.

Supplementary Figure S5

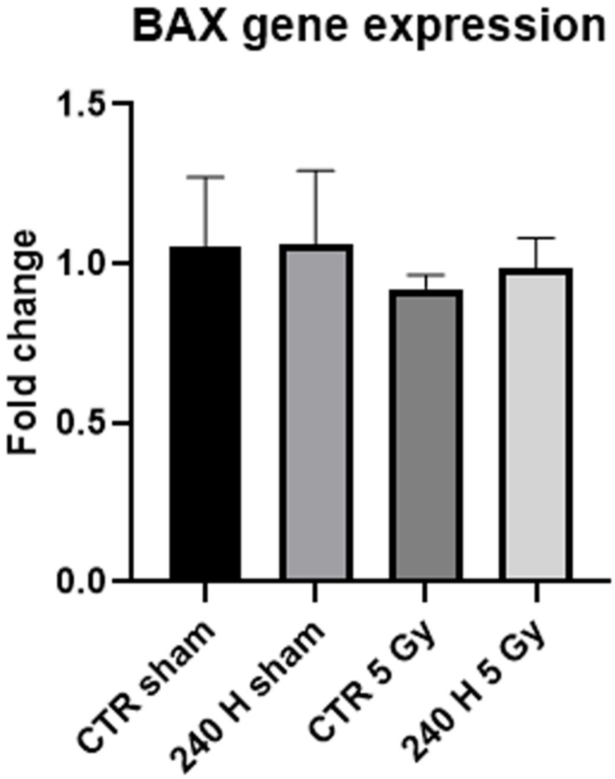

S5. BAX gene expression analysis in MB DAOY cells after 240 nm H-NDs and 1.25 MeV γ-rays combined treatment. Level of BAX mRNA was measured by quantitative real-time PCR (qPCR) in DAOY cells treated with 240 nm H-NDs and untreated (CTR) and exposed to 0 Gy (Sham) and 5 Gy (n=3) at 3 h post-irradiation. Results are shown as fold change (normalized for GAPDH). Results confirmed the absence of a significant modulation even at transcript expression level in all samples.

Dynamic Light Scattering (DLS) and Zeta Potential (ZP) Analyses

The data concerning the suspensions of OX-NDs are presented in the garph. From OX-ND size distribution by number it is possible to see that both in PBS and NaCl solution hydrodynamic size values are consistent with the dimensions of primary particles, indicating ND effective dispersion. This observations can be explainded with the marked hydrophilic character of OX-ND surface, evidenced in DRIFT spectra of Figure 1A (see main text) and dictated by the presence of oxygen-containing functionalities, which renders OX-NDs more inclined to disperse rather than aggregate [34].

ZP analyses of the OX-NDs displayed that the oxidized particles exhibit a strongly negative zeta potential both in the considered solutions. ZP was indeed equal to (-43 $\pm$ 3) mV and (-34.67 $\pm$ 0.12) mV respectively for 240 nm and 55 nm OX-NDs in PBS. Similarly, 240 nm OX-NDs have a ZP of (-25.5 $\pm$ 1.0) mV in NaCl solution, whereas 55 nm OX-NDs displayed a ZP = (-17.3 $\pm$ 1.1) mV in the same medium. Such values can be linked to the presence of surfcae oxygenated functional groups, whose acidic behavior, leading to their deprotonation, determines the formation of a markedly negative charge on the ND surface, translating in high absolute zeta potential values and reduced agglomeration tendency, due to electrostatic repulsion between particles. ZP measurements results for OX-NDs thus closely align with the size analysis.

On the other hand, H-NDs were evidenced to display pronounced agglomeration tendency, as was readily discernible even through visual inspection of samples dispersions, characterized by reduced turbidity. The significant clustering tendency, deriving from H-ND hydrophobicity (highlighted by the DRIFT data in Figure 1A of the main text), led to the formation of varying-sized aggregates, thus giving rise to very heterougeneous results in different measurements sessions. In some cases, a part of the aggregates even settled at the bottom of the solutions, resulting in a reduction of the effective ND concentration within the suspensions. Due to these experimental issues and the scarce reproducibility and solidity of the collected data, the curves related to the size distribution for H-NDs, as well as ZP results, have thus not been shown as part of our findings.

Supplementary Figure S6

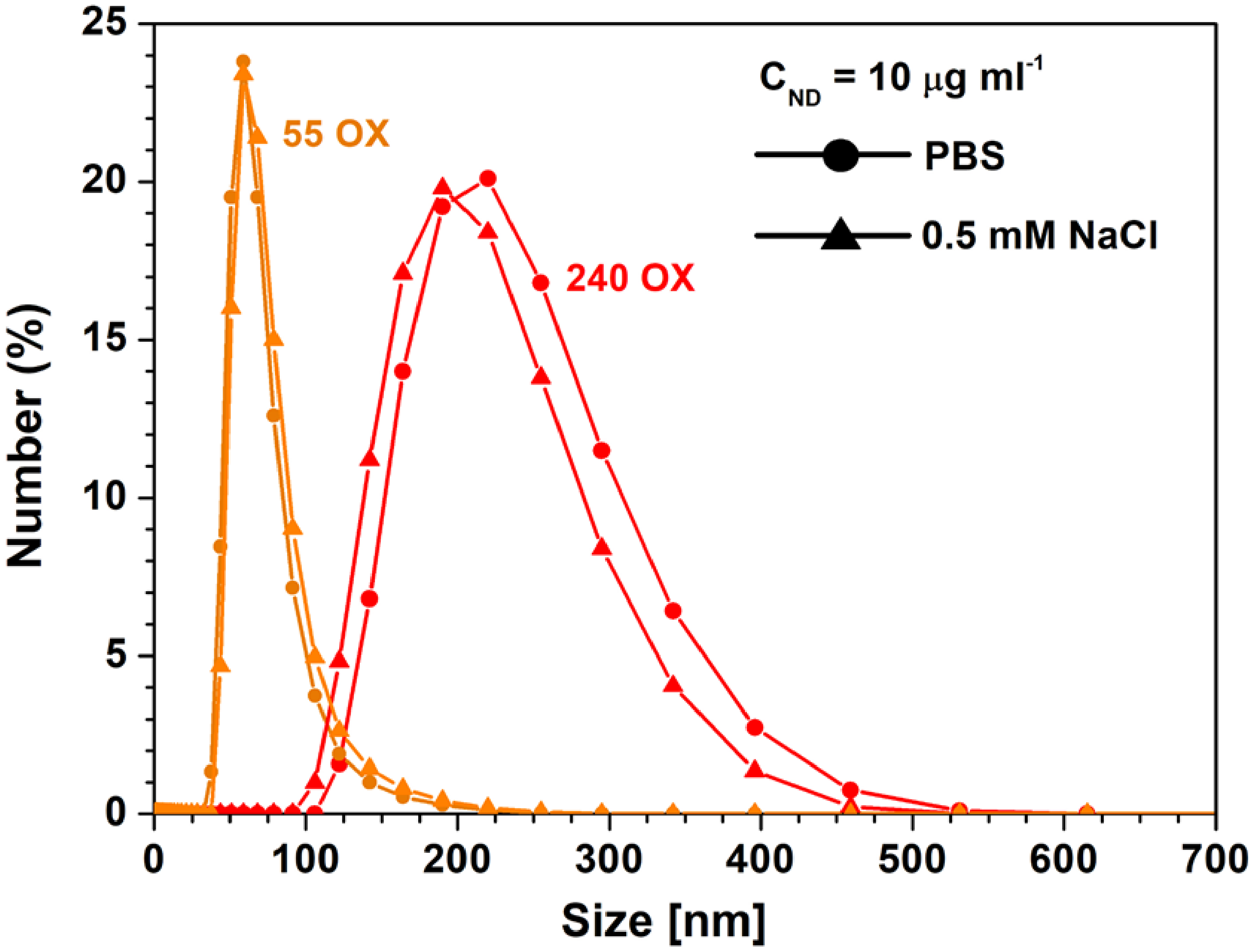

S6. OX-ND DLS size distribution by number.

References

24. Zaitsev, A.M. *Optical Properties of Diamond*; Springer, 2001.

34. Aprà, P.; Mino, L.; Battiato, A.; Olivero, P.; Sturari, S.; Valsania, M.C.; Varzi, V.; Picollo, F. Interaction of Nanodiamonds with Water: Impact of Surface Chemistry on Hydrophilicity, Aggregation and Electrical Properties. *Nanomaterials* **2021**, *11*, 2740.

59. Smith, B.R.; Gruber, D.; Plakhotnik, T. The Effects of Surface Oxidation on Luminescence of Nano Diamonds. *Diam. Relat. Mater.* **2010**, *19*, 314–318.
